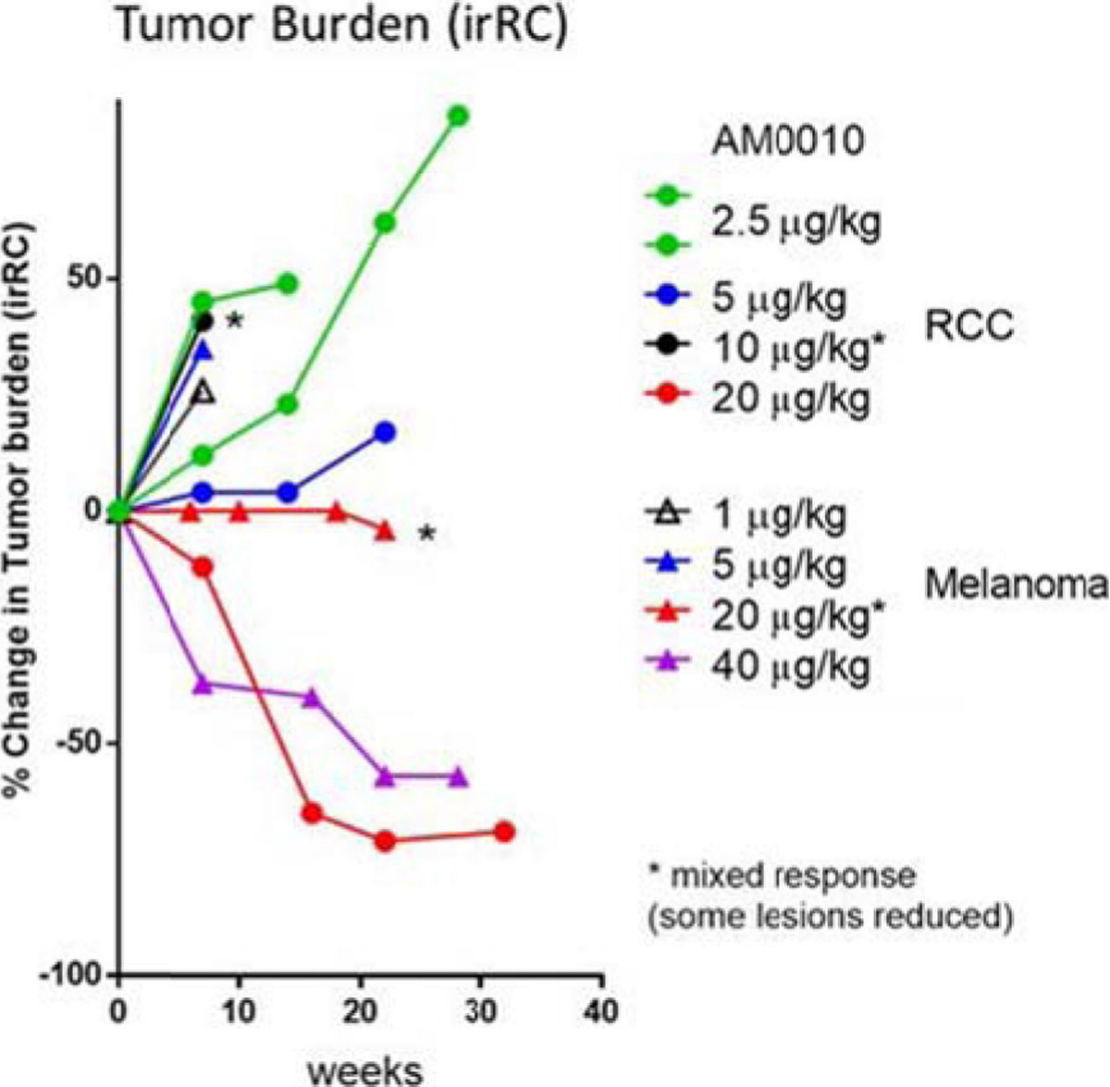# CD8+ T cell stimulation with pegylated recombinant human IL-10 in the patient with advanced solid tumors - a Phase I study

**DOI:** 10.1186/2051-1426-3-S2-P204

**Published:** 2015-11-04

**Authors:** Aung Naing, Jeffrey R Infante, Kyriakos P Papadopoulos, Karen A Autio, Deborah J Wong, Gerald S Falchook, Manish R Patel, Shubham Pant, Melinda Whiteside, Johanna Bendell, Todd M Bauer, Filip Janku, Milind Javle, Rivka Colen, Nizar Tannir, Martin Oft

**Affiliations:** 1 U.T.M.D. Anderson Cancer Center, Houston, TX, USA; 2 Sarah Cannon Research Institute / Tennessee Oncology, PLLC, Nashville, TN, USA; 3 South Texas Accelerated Research Therapeutics (START), San Antonio, TX, USA; 4 Memorial Sloan-Kettering Cancer Center, New York, NY, USA; 5 University of California Los Angeles (UCLA), Los Angeles, CA, Los Angeles, CA, USA; 6 Sarah Cannon Research Institute at HealthONE, Denver, CO, USA; 7 Florida Cancer Specialists, Sarasota, FL, USA; 8 Oklahoma University, Oklahoma City, OK, USA; 9 Armo BioSciences, Redwood City, CA, USA; 10 Sarah Cannon Research Institute/Tennessee Oncology, PLLC, Nashville, TN, USA; 11 MD Anderson, Houston, TX, USA

## Background

In preclinical studies, PEGylated IL-10 (PEG-IL-10) but not non-PEG IL-10 induces tumor rejection by the directly activating and increasing the proliferation of tumor specific CD8 T cells within the tumor. PEG-IL-10 activates STAT3 and STAT1 in tumor infiltrating CD8 T cells. PEG-IL-10 induced the expression of the Th1 signature cytokine IFNg in intratumoral CD8 T cells, which was crucial for the tumor immunity.

Concomitant application of PEG-IL-10 and chemotherapies did not disrupt the immune stimulation but lead to synergistic anti-tumor efficacy.

Phase I study with AM0010 (PEG-hIL-10) has enrolled more than 200 patients in monotherapy and in combination arms with chemotherapies, a tyrosine kinase inhibitor and a PD-1 inhibitor. Primary objectives of this study were to establish the safety, tolerability and the MTD of AM0010 as monotherapy or in combination with chemotherapies, targeted agents or immunotherapy. Secondary objectives were to assess anti-tumor-activity, pharmacokinetics, immunogenicity and AM0010 induced immune activation.

## Results

Dose escalation ranged from 1-40 mcg/kg daily in monotherapy. An MTD for monotherapy was not defined, but the recommended Phase II dose (RP2D) was defined as 20-40 mcg/kg. The half-life of AM0010 is ~20hrs. Common treatment related adverse events (AEs) included injection site reaction, rash, fatigue, thrombocytopenia and anemia. G3 adverse events in the monotherapy cohorts included anemia, thrombocytopenia, rash and fatigue. AEs promptly improved upon dose interruption and durable immune related AEs were not observed.

At RP2D, AM0010 induced a signature immune cytokine profile in the serum of all patients. It included the upregulation of cytokines IL-18 and IFNg (Th1), IL-4 and GM-CSF (dendritic cell maturation), IL-7 (T cell memory) and FasL (cytotoxic T cells). The immune suppressive cytokine TGFb was down-regulated.

The percentage of CD8 T cells positive for the activation marker PD1 increased in the blood of responding patients. Objective responses in AM0010 monotherapy were observed in 11% of immune sensitive cancers (RCC, NSCLC, melanoma). Disease control rate (DCR) across all 10 indications enrolled (including pancreatic and colorectal cancer) was 49%.

Combination with chemotherapies resulted in 78% DCR and 22% PRs across all 17 indications. Combination studies with anti-PD-1 antibodies are in progress and preliminary clinical results indicate synergistic anti-tumor activity in immune sensitive indications.

## Conclusions

AM0010 was well tolerated and lead to sustained and systemic Th1 and CD8 T cell immune stimulation in all patients. Clinical responses in monotherapy, in combination with chemotherapy and immune therapy are very encouraging and initiation of Phase II studies are planned for later this year.

**Figure 1:**